# Synthesis of ultrasmall Li–Mn spinel oxides exhibiting unusual ion exchange, electrochemical, and catalytic properties

**DOI:** 10.1038/srep15011

**Published:** 2015-10-12

**Authors:** Yumi Miyamoto, Yoshiyuki Kuroda, Tsubasa Uematsu, Hiroyuki Oshikawa, Naoya Shibata, Yuichi Ikuhara, Kosuke Suzuki, Mitsuhiro Hibino, Kazuya Yamaguchi, Noritaka Mizuno

**Affiliations:** 1 Department of Applied Chemistry, School of Engineering, The University of Tokyo, 7-3-1 Hongo, Bunkyo-ku, Tokyo 113-8656, Japan; 2 Waseda Institute for Advanced Study, Waseda University, 1-6-1 Nishiwaseda, Shinjuku-ku, Tokyo 169-8050, Japan; 3 Institute of Engineering Innovation, School of Engineering, The University of Tokyo, 2-11-16 Yayoi, Bunkyo-ku, Tokyo 113-8656, Japan

## Abstract

The efficient surface reaction and rapid ion diffusion of nanocrystalline metal oxides have prompted considerable research interest for the development of high functional materials. Herein, we present a novel low-temperature method to synthesize ultrasmall nanocrystalline spinel oxides by controlling the hydration of coexisting metal cations in an organic solvent. This method selectively led to Li–Mn spinel oxides by tuning the hydration of Li^+^ ions under mild reaction conditions (i.e., low temperature and short reaction time). These particles exhibited an ultrasmall crystallite size of 2.3 nm and a large specific surface area of 371 ± 15 m^2^ g^−1^. They exhibited unique properties such as unusual topotactic Li^+^/H^+^ ion exchange, high-rate discharge ability, and high catalytic performance for several aerobic oxidation reactions, by creating surface phenomena throughout the particles. These properties differed significantly from those of Li–Mn spinel oxides obtained by conventional solid-state methods.

Metal oxide crystal structures and compositions are expected to lead to numerous applications. Manganese-based binary oxides have attracted tremendous research interest because of their potential use as electrode materials^1^,^2^,^3^,^4^,^5^,^6^,^7^,^8^,^9^,^10^, adsorbents^11^,^12^,^13^,^14^,^15^,^16^,^17^,^18^,^19^,^20^,^21^, catalysts^22^,^23^,^24^,^25^,^26^,^27^, catalyst supports^28^,^29^, and oxidants^30^,^31^,^32^,^33^. Specifically, Li–Mn spinel oxides (LMOs) have served as cathode materials for lithium-ion batteries^1^,^2^,^3^,^4^,^5^,^6^,^7^,^8^,^9^,^10^ and Li^+^ ion-selective adsorbents^11^,^12^,^13^,^14^,^18^ because these ions can be chemically and/or electrochemically extracted from and inserted into the spinel structure. Nanocrystalline metal oxides offer large specific surface areas and short ion diffusion lengths that may contribute to the efficient surface reaction and rapid ion extraction/insertion^3^,^4^,^5^,^6^,^7^,^8^,^9^ required to achieve high functional materials. Nanocrystalline manganese oxides are typically synthesized by hydrothermal methods^5^,^10^,^34^,^35^,^36^,^37^,^38^,^39^ involving harsh conditions such as elevated temperatures, high pressures, and long reaction times, limiting control over product crystal structures and morphologies.

Manganese-based binary oxides exhibit three-dimensional frameworks, comprising MnO_6_ octahedral units and metal cations and form different crystal structures such as tunnels as well as layered and spinel structures according to the nature of the coexisting metal cations acting as templates for these structures^40^,^41^,^42^,^43^,^44^. Layered manganese-based binary oxides that consist of a large interlayer space between MnO_6_ octahedral units and hydrated cations as templates are often synthesized at relatively low temperatures^34^,^37^,^45^,^46^,^47^. On the other hand, spinel structure formation typically requires relatively high temperatures and long reaction times (Supplementary Table 1) because spaces between MnO_6_ octahedral units are too small to accommodate hydrated cations in these structures.

To control both morphologies and crystal structures, a synthetic method was designed to synthesize complex oxides such as LMO. This method assumed that spinel structures can be synthesized at low temperatures using non-hydrated metal cations as templates. Therefore, the cation hydration state was tuned by combining an organic solvent and an organic solvent-soluble manganese precursor. In this study, the rational one-pot low-temperature synthesis of LMO was achieved by controlling the Li^+^ ion hydration in an organic solvent (Fig. 1a). Amorphous Li–Mn binary oxide nanoparticles were initially prepared by room temperature reduction of tetra-*n*-butylammonium permanganate salt (TBAMnO_4_) in 2-propanol, which simultaneously acted as a reductant for MnO_4_^−^ and a solvent. Subsequently, the amorphous nanoparticles crystallized within 30 min when the temperature was increased to up to 86 °C while stirring. The method produced ultrasmall LMO particles with an average crystallite size of 2.3 nm (LMO(2.3 nm)) and an elevated average specific surface area of 371 ± 15 m^2^ g^−1^. The LMO(2.3 nm) particles showed unique characteristics such as unusual topotactic Li^+^/H^+^ ion exchange property, high-rate discharge ability applicable to cathode material for lithium-ion battery, and high catalytic performance for several oxidation reactions. These properties differed significantly from those of Li–Mn spinel oxides obtained by conventional solid-state methods (LMO(bulk)).

## Results

### Synthesis of Li–Mn spinel nanoparticles using dehydrated Li^+^ ions in an organic solvent

**Figure 1:**
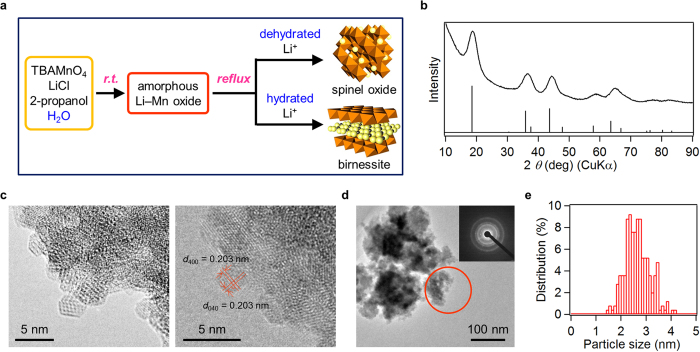
Synthesis of LMO under controlled Li^+^ ion hydration in an organic solvent. (**a**) Method concept. (**b**) XRD pattern of LMO (top) and literature data for LiMn_2_O_4_ (bottom, JCPDS 35–0782). (**c**) TEM images of LMO(2.3 nm). (**d**) TEM image of LMO(2.3 nm) over a wider area showing the region selected for electron diffraction measurements (red circle). Inset: electron diffraction pattern. (**e**) Particle size distribution of LMO(2.3 nm) calculated from the TEM images.

The low-temperature synthesis of LMO hinged on suppressing the hydration of Li^+^ ions and was consequently performed in an organic solvent using the organic solvent-soluble TBAMnO_4_ salt as an LMO precursor. TBAMnO_4_ readily dissolved in a 2-propanol-based LiCl solution to yield a purple mixture. The purple color, characteristic of MnO_4_^−^ ions, completely disappeared when the mixture was stirred for 5 min at room temperature; a brown slurry consisting of amorphous Li–Mn oxide nanoparticles was obtained. The slurry was subsequently heated at 86 °C for 30 min to generate nanoparticles. Peak positions and intensities of X-ray diffraction (XRD) pattern of these nanoparticles were in good agreement with those of LMO structures, but all observed peaks were very broad (Fig. 1b). Crystallite size estimated with Scherrer equation using width of 111 peak was 2.3 nm. On the basis of the average Mn oxidation state of 3.62 ± 0.065, which was estimated from a redox titration and the composition of LMO(2.3 nm) (Li/Mn = 0.463 (mol/mol)), which was from elemental analyses, the empirical formula of LMO(2.3 nm) was determined to be Li_0.825_(Mn_0.966_Li_0.034_)_2_O_4_. Transmission electron microscopy (TEM) images also showed that LMO(2.3 nm) consisted of small nanoparticles averaging 2.55 nm in size with a main particle size distribution of 2–3 nm (standard deviation (σ) = 19.4% (0.49 nm), Fig. 1c,e). These TEM images displayed clear lattice fringes throughout the particles (Fig. 1c). Electron diffraction and XRD patterns did not present any obvious halo pattern (Fig. 1b,d). These results indicated the crystallinity of LMO(2.3 nm) and absence of an amorphous phase in the sample. The LMO(2.3 nm) nanoparticles exhibited large Brunauer–Emmett–Teller (BET) surface areas (371 ± 15 m^2^ g^−1^), consistent with their small size. These observations demonstrate that mild synthetic conditions (i.e., low temperature and short reaction time) have led to LMO(2.3 nm) with the ultrasmall particle sizes. These nanoparticles displayed smaller sizes and larger specific surface areas than previously reported LMOs (Supplementary Table 1).

Due to the ultrasmall particle size and the large specific surface areas of LMO(2.3 nm), the surface structure of LMO(2.3 nm) is expected to be different from those of LMOs with large particle sizes. Thus, we next performed the surface characterization of LMO(2.3 nm) and LMO(bulk). LMO(bulk) exhibited an average particle size of 410 nm (according to the BET surface area). The average oxidation states of LMO(2.3 nm) and LMO(bulk) were 3.62 ± 0.065 and 3.48 ± 0.018, respectively, indicative of the mixed valency of Mn. Electron paramagnetic resonance (EPR) spectra of LMO(2.3 nm) showed only a very broad and low intensity peak, and the characteristic six-peak hyperfine structures due to Mn^2+^ species were not observed (Supplementary Fig. 1). Thus, LMOs are predominantly composed of Mn^4+^ and Mn^3+^ species. The XPS spectra of LMO in the Mn 2p region showed no significant difference between LMO(2.3 nm) and LMO(bulk) (Supplementary Fig. 2). This indicated that the relative concentration of Mn^4+^ (Mn^3+^) species on the surface of LMO(2.3 nm) was almost the same as that of LMO(bulk). On the other hand, the XPS spectrum of LMO(2.3 nm) in the O 1s region was much different from that of LMO(bulk) (Supplementary Fig. 3). Each O 1 s spectrum can be deconvoluted into three peaks corresponding to the three types of oxygen species; the low (around 530 eV), medium (around 531 eV), and high binding energy peaks (around 533 eV) are possibly ascribed to the coordinatively saturated oxygen species (lattice oxygen, described as O_sat_), the coordinatively unsaturated oxygen species (surface adsorbed oxygen, OH groups on the surface, or oxygen vacancy, described as O_unsat_), and adsorbed H_2_O molecule, respectively^48^. The curve-fitting analyses showed that the O_unsat_/(O_sat_ + O_unsat_) values for LMO(2.3 nm) and LMO(bulk) were 0.38 and 0.13, respectively, indicating that the Mn and O species on the surface of LMO(2.3 nm) are in lower coordination environments in comparison with those of LMO(bulk). This may contribute to the enhancement of the surface reactivity of LMO(2.3 nm) (e.g. catalytic reactions).

**Figure 2:**
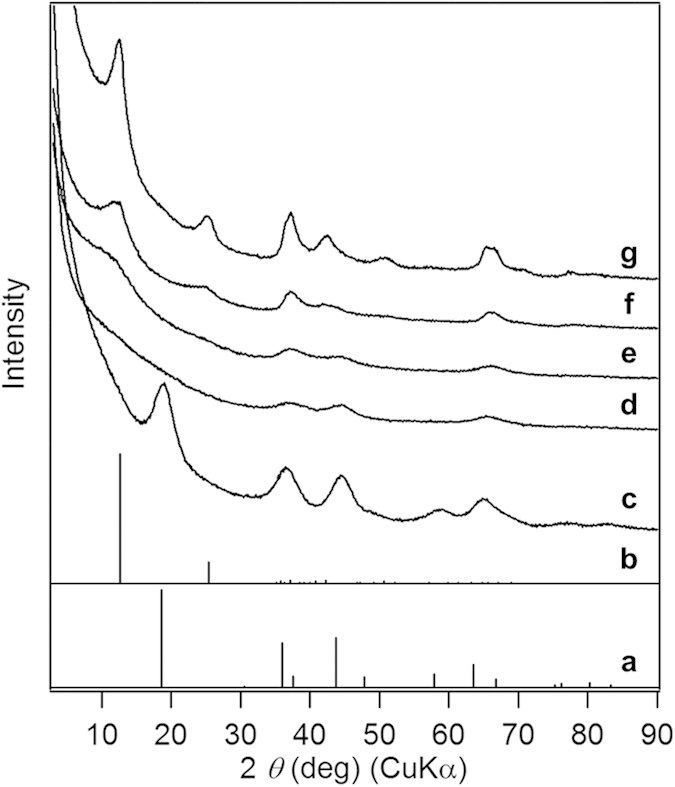
The effect of water addition to organic solvents on product phases. Literature data for (**a**) LiMn_2_O_4_ (JCPDS 35-0782) and (**b**) Li-birnessite (JCPDS 50-0009). XRD patterns of products synthesized using H_2_O/2-propanol mixtures as solvents ((**c**) H_2_O/Li molar ratios of 0, (**d**) 10, (**e**) 20, (**f**) 200, and (**g**) 500).

This low-temperature synthesis of nanocrystalline LMO assumed the effectiveness of water exclusion using an organic solvent. To assess its effect on the product structure, water was incrementally added to the solvent during the synthesis of nanocrystalline LMO. Spinel phase was predominantly formed only in the absence of water (H_2_O/Li ≈ 0 (mol/mol)) (Fig. 2c). Diffraction peaks characteristic of the spinel structure disappeared when a specific amount of water corresponding to a H_2_O/Li molar ratio of 10 was added to the solvent (Fig. 2d). The product showed diffraction peaks assignable to the birnessite-type layered manganese oxide when the H_2_O/Li molar ratio increased up to 20 (Fig. 2e). Further increases in water amounts (H_2_O/Li = 200 or 500 (mol/mol)) afforded the well-developed birnessite-type structure (Fig. 2f,g). These results clearly show that product phases can be controlled by manipulating the amounts of water added to the solvent. In spinel structures, Li^+^ ions occupy tetrahedral sites surrounded by closely packed O^2–^ species. Therefore, highly hydrated Li^+^ ions likely present relatively large hydration radii, preventing their incorporation into spinel structures. In contrast, these highly hydrated Li^+^ ions are readily integrated into layered structures displaying a larger interlayer space. This suggests that a precise control of the Li^+^ ion hydration in organic solvents is essential for the low-temperature synthesis of nanocrystalline LMOs. To the best of our knowledge, this is the first low-temperature nanocrystalline spinel oxide synthesis achieved by the concept of controlling the hydration of coexisting metal cations.

**Figure 3:**
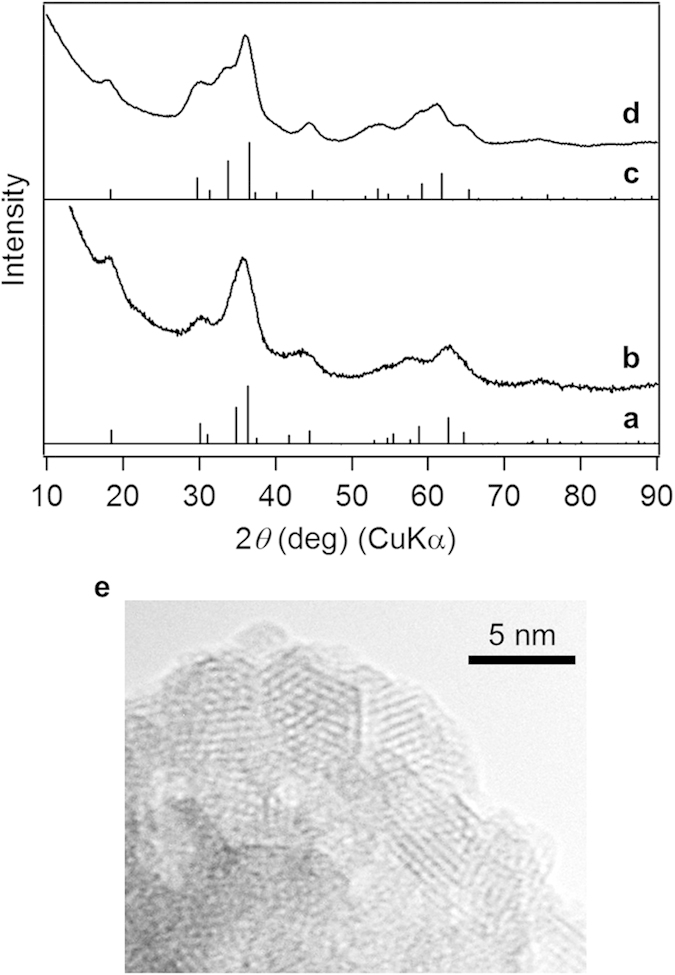
Synthesis of CMO and ZMO under controlled Li^+^ ion hydration in an organic solvent. (**a**) Simulated pattern of CMO, (**b**) XRD pattern of CMO, (**c**) simulated pattern of ZMO, and (**d**) XRD pattern of ZMO. (**e**) TEM image of CMO.

Furthermore, this low-water approach using TBAMnO_4_ in 2-propanol could be applied to the syntheses of other manganese-based binary spinel nanoparticles such as Co–Mn spinel oxides (CMOs) and Zn–Mn spinel oxides (ZMOs) by using CoCl_2_ or ZnCl_2_ instead of LiCl (Fig. 3). The products showed the XRD patterns assignable to the CMO and ZMO structures (Fig. 3b,d). The CMO and ZMO nanoparticles obtained by the present procedure also showed the small crystallite sizes (CMO: 2.78 nm, ZMO: 2.97 nm) and the large BET surface areas (CMO: 257 m^2^ g^−1^, ZMO: 110 m^2^ g^−1^). The TEM image of CMO indicated that CMO consisted of the small crystalline nanoparticles (Fig. 3e).

These results revealed that the crystallization into the spinel phase does not require a temperature as high as that previously employed (higher than 180 °C in most cases, Supplementary Table 1). The use of an organic solvent canceled the energetic demands related to the coexisting metal cation (e.g. Li^+^, Co^2+^, and Zn^2+^) dehydration and structural transformation of birnessite-type layered intermediates into the spinel phase to possibly reveal the actual requirements of the crystallization. This understanding enabled temperature and reaction time optimization to give the smallest manganese-based binary spinel nanoparticles, which may be applicable to a wide range of (binary) metal oxides.

### Specific ion exchange properties of Li–Mn spinel nanoparticles

**Figure 4:**
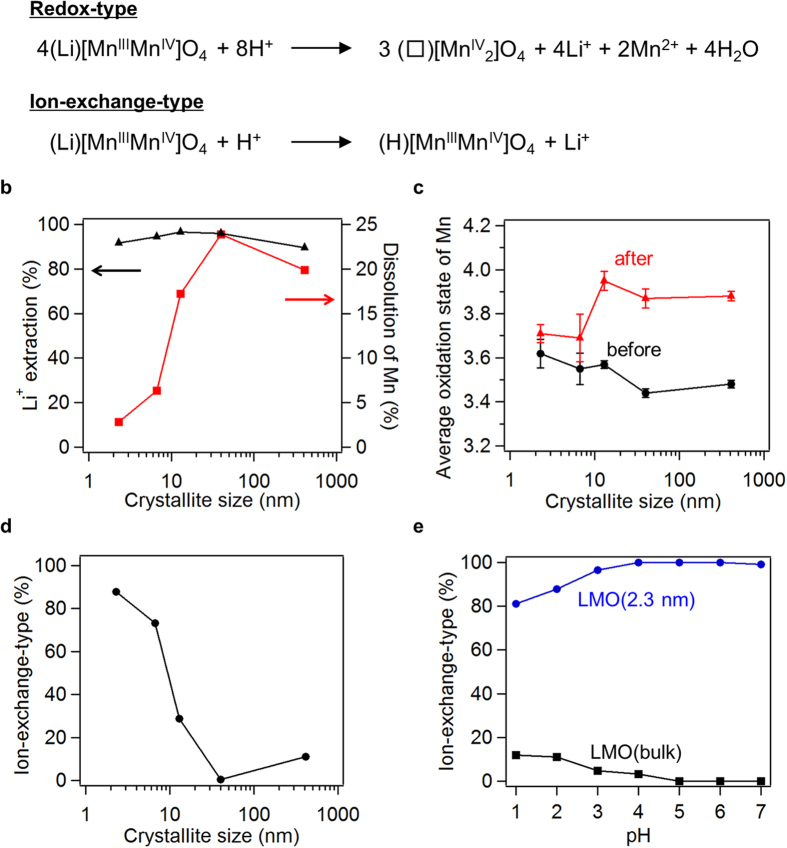
Li^+^ ion extraction from LMO under acidic conditions. All reactions were carried out for 30 min. We confirmed that all reactions reached their equilibria within 30 min. (**a**) Li^+^ ion extraction reactions from LMO in an acid aqueous solution through redox or ion exchange reaction. (**b**) Li^+^ ion extraction (black) and Mn dissolution ratios (red) for LMOs stirred in nitric aqueous solution (pH 2) at room temperature as a function of particle size. (**c**) Average oxidation states of Mn in LMOs before (black) and after acid treatment (red). (**d**) Li^+^ ion extraction by ion exchange reaction. (**e**) Ion exchange reaction as a function of pH. Data shown in (**b**), (**c**), and (**d**) are plotted against crystallite sizes for LMO(2.3 nm), LMO(6.7 nm), LMO(13 nm), and LMO(40 nm) and against particle sizes calculated from the BET surface area for LMO(bulk). In (**d**) and (**e**), “ion-exchange-type” means the contribution of ion exchange reaction to Li^+^ ion extraction reaction from LMOs. (**e**) For LMO(bulk), the Li^+^ ion extraction hardly proceeded (Supplementary Table 4) under weakly acidic conditions (pH > 5). At pH 7, LMOs were dispersed in neutral water without pH adjustment.

In general, Li^+^ ions can be extracted from LMOs with aqueous acidic solutions to produce a spinel-type λ-manganese oxide that acts as an Li^+^ ion-selective adsorbent^11^,^12^,^13^,^14^,^18^. This acid-mediated extraction from bulk LMO predominantly involves a redox reaction, concomitant with the disproportionation of Mn^3+^ species, resulting in dissolution of formed Mn^2+^ species and increasing the average oxidation state of the oxide manganese species (Fig. 4a, upper)^14^. Alternatively, it was reported that an Li^+^/H^+^ ion exchange reaction is possible at the particle surfaces (Fig. 4a, lower)^49^. Therefore, surface reactions may proceed throughout the particle upon LMO particle size reduction.

The acid-mediated Li^+^ ion extraction was investigated for LMO samples with various particle sizes (Supplementary Fig. 4 and Supplementary Table 2). LMO samples were dispersed in an aqueous nitric acid at pH 2 and stirred for 30 min at room temperature. This acid treatment extracted about 90% of Li^+^ ions from all spinel structures (Fig. 4b and Supplementary Fig. 5). We confirmed that all reactions reached their equilibria within 30 min. The redox reaction was predominant in LMO(bulk), which exhibited an average particle size of 410 nm (according to the BET surface area), and LMO(40 nm) (Fig. 4b–d). This resulted in the dissolution of the manganese species and the increase of the average oxidation state, which were effectively suppressed in LMO samples exhibiting smaller particle sizes (Fig. 4b–d). Consequently, the topotactic ion exchange reaction may proceed in smaller LMO nanoparticles.

The dissolution of manganese species and the increase of the average oxidation state hardly occurred in LMO(2.3 nm), indicating that the Li^+^ ion extraction from the spinel structure proceeded exclusively by ion exchange (Fig. 4b–d). EPR analysis revealed the absence of Mn^2+^ species in the LMO(2.3 nm) sample retrieved after the Li^+^ ion extraction (Supplementary Fig. 1). In addition, the Li^+^ extraction mainly followed this ion exchange process under all pH conditions examined (pH 1–7) and was almost quantitative above pH 4 for LMO(2.3 nm) (Fig. 4e, Supplementary Figs 6, 7 and Supplementary Table 4). In contrast, it mainly proceeded by redox reaction under all pH conditions but hardly occurred above pH 5 for LMO(bulk) (Fig. 4e, Supplementary Figs 6, 7 and Supplementary Table 4). Interestingly, 19% of Li^+^ ions were extracted from LMO(2.3 nm) by ion exchange reaction even when dispersed in neutral deionized water (30% extraction was possible after two extraction cycles) but to a small extent from LMO(bulk) under the same conditions (Supplementary Figs 6, 7 and Supplementary Table 4). Therefore, unlike LMO(bulk), LMO(2.3 nm) abstracted H^+^ from even neutral water (Supplementary Figs 6, 7 and Supplementary Table 4). In the presence of a large amount of aqueous nitric acid (1000 mL for 10 mg Li–Mn spinel oxide), more than 90% of Li^+^ was extracted from LMO(2.3 nm) by more than 99% ion exchange reaction under even at the relatively high pH condition (pH 5) (Supplementary Fig. 8 and Supplementary Table 5). Only 39% Li^+^ extraction, obtained by almost 100% redox reaction, was observed for LMO(bulk) under the same conditions (Supplementary Fig. 8 and Supplementary Table 5). After extraction, Li^+^ ions were readily re-inserted into the LMO(2.3 nm) spinel structure by dispersion in 0.1 M LiCl–LiOH aqueous solution at room temperature (Supplementary Fig. 9 and Supplementary Table 6). Therefore, when the LMO particle size decreased, mass transfer such as the ion exchange reaction became more dominant than the redox reaction, resulting in the unusual topotactic Li^+^–H^+^ ion exchange observed in LMO(2.3 nm).

### Electrochemical properties of Li–Mn spinel nanoparticles

**Figure 5:**
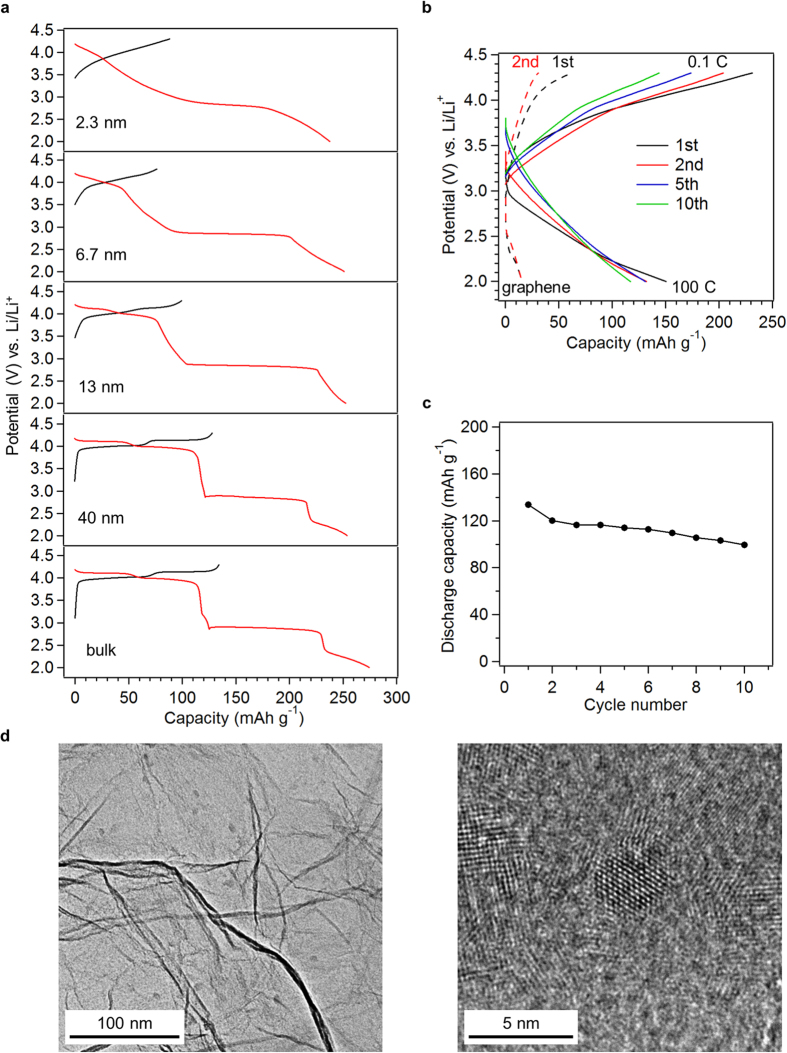
Electrochemical properties of LMO. (**a**) First charge (black) and discharge (red) curves of LMO with crystallite sizes of 2.3, 6.7, 13, and 40 nm and LMO(bulk) at a constant charge and discharge rate of 0.1 C (14.8 mA g^−1^) between 2 and 4.3 V. (**b**) Charge and discharge curves of LMO–G. The cell was fully charged to 4.3 V at 0.1 C and subsequently discharged to 2 V at 100 C. (**c**) Discharge capacity of LMO in LMO–G at 100 C. The cell was fully charged to 4.3 V at 0.1 C and subsequently discharged to 2 V at 100 C. Charge and discharge capacities were calculated based on the weight of LMO by subtracting the graphene capacity from the LMO–G capacity. The graphene capacity in LMO–G was estimated by electrochemical tests performed on graphene electrode under the corresponding charge and discharge conditions. (**d**) TEM images of LMO–G at (left) low and (right) high magnifications.

Because of the possible electrochemical Li^+^ extraction from and insertion into its spinel structure, LMO has attracted significant interest as a cathode material for lithium ion batteries^1^,^2^,^3^,^4^,^5^,^6^,^7^,^8^,^9^,^10^. The electrochemical properties of LMO particles in different sizes (Supplementary Fig. 4 and Supplementary Table 2) were investigated by collecting their voltage curves during charge and discharge corresponding to Li^+^ ion extraction and insertion processes, respectively. The charge/discharge rate amounted to 0.1 C (1 C = 148 mA g^−1^). Typical charge/discharge curves of LMOs show plateau regions at around 4 V (region I) and 3  V (region II)^50^. Plateau regions I and II correspond to the Li^+^ insertion into (or extraction from) the spinel structure and biphasic region comprising spinel and rock-salt structures, respectively. The area below region II corresponds to the Li^+^ insertion into (or extraction from) the rock-salt structure^50^. Figure 5a shows the LMO charge/discharge voltage curves for a first charge reaching 4.3 V. Smaller LMO particles presented smaller discharge capacities in the plateau region approximating 4 V and larger capacities below this plateau (Fig. 5a). While this tendency associated with the cathode materials size reduction was consistent with the previous studies^5^,^51^, region I was almost undetectable in LMO(2.3 nm) charge/discharge curves. The Li^+^ site energy distribution, which is usually only within a few nanometers of conventional particle surfaces^52^,^53^, appeared throughout the LMO(2.3 nm) particle. As a result, voltage changes stemmed from the variations in the site energy distribution (Supplementary Fig. 10). The existence of plateau at around 3 V (region II) suggests that excess Li^+^ inserted into the spinel structure via biphasic spinel/rock-salt state formation (Fig. 5a).

The short Li^+^-diffusion length and high electrochemical surface area of the nanoparticles are expected to impart high discharge rate capability to the resulting electrode. However, the tendency of LMO(2.3 nm) to form dense aggregates (Supplementary Fig. 11) may resulting in high electrode resistance, limiting electrochemical performance such as discharge rate capabilities. In fact, LMO(2.3 nm) displayed smaller first charge/discharge capacities than theoretical values (theoretical charge capacity: 148 mAh g^−1^, theoretical discharge capacity: 296 mAh g^−1^) (Fig. 5a). To improve the electrochemical property of LMO(2.3 nm), a Li–Mn spinel nanoparticles–graphene composite (LMO–G) was prepared by adding graphene to the LMO synthetic solution to prevent LMO particle aggregation and promote electronic conduction (Fig. 5d and Supplementary Fig. 12). Electrochemical performance improvements were assessed by measuring the discharge rate of LMO–G. Charge and discharge capacities were expressed based on the LMO weight in the composite after subtracting the graphene contribution from the observed capacities. Figure 5b demonstrated that LMO delivered an extremely high discharge capacity of 134 mAh g^−1^ even at 100 C. Electrode elemental analyses provided a Li/Mn molar ratio of 0.37 for Li^+^ ion insertion during the first discharge at 100 C, consistent with that calculated from the LMO discharge capacity (Li/Mn = 0.45  (mol/mol)). The difference between these ratios may result from the electric double layer capacitance contribution of the high-surface-area LMO to the discharge capacity. No plateau was observed for the spinel/rock-salt biphasic region in Fig. 5b. Therefore, the LMO–G discharge at 100 C corresponded to Li^+^ ion insertion into the spinel structure without transformation into a rock-salt structure although the cell was discharged to 2 V. This originated from the large ohmic resistance-related voltage drop due to such high current density, as indicated by the low initial voltage of the discharge curves (Fig. 5b). Hence, LMO–G did not undergo any phase transition, which often causes poor cycle durability, and exhibited good capacity retention (Fig. 5c). These high discharge rate capabilities arose from the short Li^+^-diffusion length and high electrochemical surface area of the small LMO particles.

### Catalytic performance of Li–Mn spinel nanoparticles

**Figure 6:**
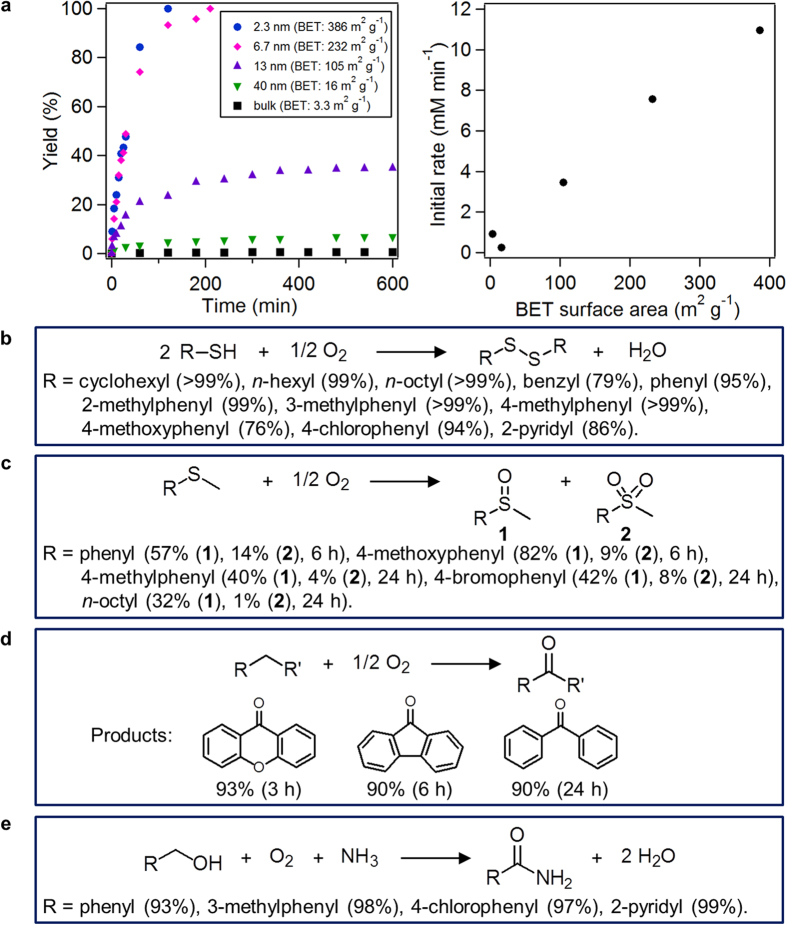
Catalytic activities of LMOs for oxidation reactions. (**a**) Reaction profiles (left) and initial rates (right) for cyclohexanethiol homocoupling in the presence of LMO exhibiting different particle sizes. Reaction conditions: LMO (20 mg), substrate (1.25 mmol), acetonitrile (5 mL), 0 °C, air (1 atm). (**b**) LMO(2.3 nm)-catalyzed oxidative thiol homocoupling. Reaction conditions: LMO(2.3 nm) (20 mg), substrate (0.25 mmol), acetonitrile (1 mL), 30 °C, O_2_ (1 atm), 1 min. (**c**) LMO(2.3 nm)-catalyzed sulfide oxidation. Reaction conditions: LMO(2.3 nm) (50 mg), substrate (0.5 mmol), *o*-dichlorobenzene (1 mL), 150 °C, O_2_ (5 atm). (**d**) LMO(2.3 nm)-catalyzed alkylarene oxidation. Reaction conditions: LMO(2.3 nm) (50 mg), substrate (0.5 mmol), *o*-dichlorobenzene (1 mL), 150 °C, O_2_ (5 atm). (**e**) LMO(2.3 nm)-catalyzed oxidative amidation. Reaction conditions: LMO(2.3 nm) (50 mg), substrate (0.25 mmol), 28% aqueous ammonia (50 μL), 1,4-dioxane (1 mL), 150 °C, O_2_ (3 atm), 1 h. Product yields were determined by gas chromatography using naphthalene as an internal standard.

Because Mn adopts various oxidation states, its oxides can act as oxidants^30^,^31^,^32^,^33^ and catalysts^22^,^23^,^24^,^25^,^26^,^27^ for several oxidative functional group transformations. The catalytic activities of different LMO particles (Supplementary Fig. 4 and Supplementary Table 2) were initially investigated for the oxidative homocoupling of cyclohexanethiol into dicyclohexyldisulfide at 0 °C. Nanoparticles displaying the smallest particle sizes and highest BET surface areas exhibited the highest catalytic activity for the homocoupling (Fig. 6a). Initial reaction rates were almost proportional to the nanoparticles BET surface areas (Fig. 6a). On the other hand, when the reaction time was prolonged, differences in catalytic activities increased depending on particle size. Dicyclohexyldisulfide was obtained in only 0.66% yield after 10 h using LMO(bulk) whereas the reaction was completed within 2 h in the presence of LMO(2.3 nm) under the conditions described in Fig. 6a. A smooth redox reaction of the metal (in particular, re-oxidation of the reduced metal by O_2_ species) becomes important in manganese-based oxide-catalyzed transformations. When catalyst particle sizes decrease and BET surface areas increase, the influence of surface energy may become considerable and the surface metal species are in low coordination environments. The redox equilibrium of metal oxides changes according to their particle size because of particle surface energy effects^54^. This study assumed that the considerable catalytic activity of LMO(2.3 nm) resulted from the smooth redox reaction of Mn after the initial reactions on LMO surfaces.

Under the optimized reaction conditions, the LMO(2.3 nm) particles acted as an efficient homocoupling catalyst for various kinds of structurally diverse thiols including alkyl, aromatic, and heteroaromatic ones, typically selectively producing the corresponding disulfides in high yields within only 1 min (Fig. 6b). In addition, they could be reused at least twice for the homocoupling of cyclohexanethiol to give dicyclohexyldisulfide in high yields (yields for the first reuse: 99%, the second reuse: 95%) and retained their spinel structure after these experiments (Supplementary Fig. 13). To date, several efficient catalytic systems for aerobic oxidative homocoupling of thiols have been developed. In comparison with the previously reported catalysts, the present LMO(2.3 nm) catalyst possesses several noteworthy features; for example, (i) use of recyclable LMO(2.3 nm), (ii) simple operations, (iii) use of the greenest oxidant of O_2_, (iv) wide substrate scope, (v) mild reaction conditions, and/or (vi) very short reaction times.

Furthermore, LMO(2.3 nm) could catalyze aerobic oxygenation of various sulfides and alkylarenes (Fig. 6c,d). Oxygenation of sulfides to sulfoxides and/or sulfones is an important reaction, and catalytic oxygenation of sulfides has generally been performed by using hydrogen peroxide or *tert*-butyl hydroperoxide as the oxidant. Although several homogeneously catalyzed systems for aerobic oxygenation of sulfides have been reported, to date there have been only a few reports on heterogeneously catalyzed aerobic oxygenation of sulfides.

Finally, we demonstrated the use of LMO(2.3 nm) for oxidative amidation of primary alcohols to primary amides using O_2_ as the oxidant and aqueous NH_3_ as the nitrogen source. The oxidative amidation is a recently developed reaction by us and composed of the following four relay steps; (i) oxidative dehydrogenation of primary alcohols to aldehydes, (ii) dehydrative condensation of the aldehydes with NH_3_ to aldimines, (iii) oxidative dehydrogenation of the aldimines to nitriles, and (iv) hydration of the nitriles to form the corresponding primary amides^25^,^26^. In order to realize the oxidative amidation as the efficient one-pot procedure, both oxidation and hydration abilities should be needed for the catalysts, and we utilize a manganese oxide octahedral molecular sieve (OMS-2) as the catalyst in the previous study^25^,^26^. Notably, in the presence of LMO(2.3 nm), various kinds of primary alcohols could quantitatively be converted into the corresponding primary amides within 1 h (Fig. 6e). It typically required at least 3 h to complete these reactions when using OMS-2. Under the present conditions, LMO(bulk) did not produce benzamide at all (benzamide: <1%, benzaldehyde: 8%). The high performance of LMO(2.3 nm) is likely due to the smooth redox property (for oxidation) and the surface coordinatively unsaturated oxygen species (for hydration). The catalysis of LMO(2.3 nm) was truly heterogeneous, and LMO(2.3 nm) could be reused for the oxidative amidation of benzyl alcohol without an appreciable loss of its catalytic performance; for the reuse experiment, benzamide was obtained in 98% yield.

## Discussion

In summary, a novel low-temperature method was developed to synthesize Li–Mn spinel oxide (LMO) by suppressing the hydration of Li^+^ in an organic solvent. This mild procedure produced ultrasmall LMO particles and moreover was applicable to other manganese-based binary spinel nanoparticles such as Co–Mn spinel oxides and Zn–Mn spinel oxides. The product phase was controlled by tuning the amount of water added to the solvent, giving birnessite in the presence of large water quantities. The LMO(2.3 nm) particles exhibited unusual ion exchange between Li^+^ and H^+^ in addition to specific charge/discharge curves because of their Li^+^ site energy distribution. Furthermore, their combination with graphene led to a cathode material displaying high discharge rate capability. The properties of LMO(2.3 nm) are much more significant than those expected simply by the increase in the surface area, which is due to uniqueness of surfaces for insertion–extraction materials. Because the present synthetic method is specifically useful for the size control of insertion–extraction materials, controlling the hydration state of the templates, these findings are quite meaningful as a novel concept for the development of highly functional insertion–extraction materials. Also, LMO(2.3 nm) effectively catalyzed several aerobic oxidation reactions, such as homocoupling of thiols to disulfides, oxygenation of sulfides to sulfoxides and sulfones, oxygenation of alkylarenes to ketones, and oxidative amidation of primary alcohols to primary amides. These results demonstrated that the new approach provided ultrasmall LMOs exhibiting unique properties, such as ion exchangeabilities, electrochemical properties, and catalytic activities, by creating surface phenomenon throughout the particles. This method offers structural control and functional design for various metal oxide nanoparticles at low temperatures.

## Methods

### Materials

KMnO_4_, 2-propanol, ethanol, acetone, nitric acid, ethylene carbonate (EC), and dimethyl carbonate (DMC) were purchased from Kanto Chemical. Tetrabutylammonium bromide (TBABr) was purchased from TCI. LiCl, MnCO_3_•nH_2_O, Li_2_CO_3_, and naphthalene were acquired from Wako. Graphene was obtained from Graphene Laboratories, Inc. The 1 M LiPF_6_ solution in 1:1 EC/DMC (v/v) was acquired from Kishida. Solvents and substrates for thiol homocoupling, sulfide oxidation, alkylarene oxidation, and oxidative amidation were purchased from Kanto, TCI, Wako, and Aldrich. All reagents were used as received without purification.

### Instruments

X-ray diffraction (XRD) patterns were recorded using a Rigaku SmartLab instrument under CuKα radiation (λ = 1.5418 Å, 45 kV, 200 mA). Elemental analyses were performed by inductively coupled plasma–atomic emission spectroscopy using a Shimadzu ICPS-8100 apparatus. The average oxidation states of Mn were determined by redox titration, during which Mn in the sample was reduced with excess Fe(NH_4_)_2_(SO_4_)_2_ and unreacted Fe^2+^ was titrated with KMnO_4_ aqueous solution. This redox titration was repeated three times for each sample and average oxidation states were defined as average value ± standard deviation. Brunauer–Emmett–Teller (BET) surface areas were measured by N_2_ adsorption at −196 °C using a micromeritics ASAP 2010 instrument. Transmission electron microscopy (TEM) images were obtained using JEOL JEM-2800 or JEM-2000EX II at an acceleration voltage of 200 kV. TEM specimens were prepared by dispersing the LMOs in ethanol and sonicating them for 30 min before deposition on carbon-coated copper grid. Scanning electron microscopy (SEM) images were obtained using Hitachi S-4700 at an acceleration voltage of 15 kV. SEM samples were prepared by dispersing the LMOs in ethanol and depositing them on carbon-coated copper grid. X-ray photoelectron spectroscopy (XPS) analyses were performed using a JEOL JPS-9000 under Mg Kα radiation (*hν*  = 1253.6 eV, 8 kV, 10 mA). The peak positions were calibrated by C 1s peak of trace amount of carbon impurities (284.0 eV). The baselines were subtracted by the Shirley method. Electron paramagnetic resonance (EPR) analyses were performed at −100 °C using a JEOL JES-RE-1X. Gas chromatography analyses were performed using a Shimadzu GC-2014 apparatus equipped with a flame ionization detector.

### Synthesis of TBAMnO_4_

An aqueous TBABr solution (0.4 M, 50 mL) was slowly added to an aqueous KMnO_4_ solution (0.4 M, 50 mL) under vigorous stirring at room temperature to form purple precipitates. The slurry containing the precipitates was further stirred for 3 h at room temperature. The precipitates were collected by filtration under reduced pressure, washed with deionized water several times and dried under reduced pressure overnight at room temperature. Caution: TBAMnO_4_ can react violently with itself (MnO_4_^−^ can oxidize the C–H bonds of the TBA cation) and possibly catch fire. Thus, it should be treated carefully and stored in the appropriate condition (e.g. refrigerated conditions).

### Synthesis of LMO(2.3 nm)

TBAMnO_4_ (1.50 mmol) was added to a LiCl solution (2 M, 50 mL) in 2-propanol and stirred at room temperature for 5 min to give a brown precipitate. The resulting slurry was subsequently heated to 86 °C and stirred at 86 °C for 30 min. The precipitates were collected by membrane filtration (average pore size: 0.2 μm) under reduced pressure and successively washed with deionized water (ca. 100 mL) and acetone before drying at 120 °C.

### Effect of water on the product phase

The effect of water on the product phase was investigated using 2-propanol/H_2_O mixtures as solvents for reaction time of 3 h. Synthetic solution compositions are shown in Supplementary Table 7.

### Synthesis of CMO and ZMO

TBAMnO_4_ (0.15 mmol) was added to a LiCl solution (0.015 M, 5 mL) in 2-propanol and stirred at room temperature for 5 min to give a brown precipitate. The resulting slurry was subsequently heated to ca. 82 °C and stirred at 82 °C for 12 h. The precipitates were collected by membrane filtration (average pore size: 0.2 μm) under reduced pressure and successively washed with deionized water and acetone before drying at 120 °C.

### Synthesis of LMO with different particle sizes

LMO(6.7 nm), LMO(13 nm), and LMO(40 nm) were synthesized by a hydrothermal method^38^. The organic reducing agent (1.1 equiv. to Mn) was added to a mixture (91 mL) containing LiOH (0.1 M) and KMnO_4_ (0.077 M) and the solution was stirred at room temperature for 1 min. The organic reducing agent was ethanol for LMO(6.7 nm) and acetone for LMO(13 nm) and LMO(40 nm). The reaction mixture was transferred into a polytetrafluoroethylene (PTFE)-lined stainless steel autoclave and heated at 180 °C for 5 h to give LMO(6.7 nm) and LMO(13 nm) or 72 h to generate LMO(40 nm). Precipitates were collected by membrane filtration (average pore size: 0.2 μm) under reduced pressure, washed with deionized water and dried at 120 °C overnight. LMO(bulk) was synthesized by a conventional solid state method. MnCO_3_•nH_2_O and Li_2_CO_3_ were mixed and ground in an agate mortar (Li/Mn molar ratio = 1:2) before successive calcinations at 700 °C for 21 h and 825 °C for 21 h.

### Li^+^ ion extraction from LMO in aqueous acid solutions

Li–Mn spinel oxide (200 mg) was added to an aqueous nitric acid solution (40 mL) and the solution pH was kept constant for 30 min at room temperature. We confirmed that all reactions in Fig. 4 reached their equilibria within 30 min. The products were collected by membrane filtration (average pore size: 0.2 μm) under reduced pressure, washed with deionized water several times, and dried at 120 °C overnight. Then, the products were characterized.

### Li^+^ ion insertion into λ-MnO_2_ nanoparticles

λ-MnO_2_ nanoparticles obtained by extracting Li^+^ ions from LMO by acid treatment at pH 2 (30 mg) were added to a mixture of LiCl (0.1 M) and LiOH·H_2_O (0.1 M) and stirred at room temperature for 30 min or 6 h. The product was collected by membrane filtration (average pore size: 0.2 μm) under reduced pressure, washed with deionized water several times, and dried at 120 °C overnight. Then, the products were characterized.

### Synthesis of LMO–G

Graphene powder (28 mg) was dispersed in 2 M LiCl in 2-propanol (10 mL) and TBAMnO_4_ was added to the solution at room temperature. Subsequently, the slurry was stirred at room temperature for 5 min and heated at 86 °C for 30 min. The resulting precipitates were collected by membrane filtration (average pore size: 0.2 μm) under reduced pressure and washed with deionized water (ca. 30 mL) and acetone before drying at 120 °C overnight. The amount of LMO in LMO–G was estimated by elemental analysis.

### Electrochemical measurements of LMO

Cathodes were prepared by mixing LMO (or LMO–G) with acetylene black and PTFE according to compositions shown in Supplementary Table 8. The mixture was pressed on an Al-mesh (100 mesh) current collector and the resulting electrode was dried at 120 °C overnight prior to use. Lithium metal was used as an anode while 1 M LiPF_6_ in 1:1 EC/DMC (v/v) acted as electrolyte solution. The 2032-type coin cells were assembled from the LMO-based cathode, lithium metal anode, electrolyte solution, glass filter as a separator, and metallic lithium in an Ar-filled glove box. Cells were charged to 4.3 V at a current density of 0.1 C and discharged to 2.0 V at required current densities (0.1 or 100 C). The current density of 1 C rate was defined as 148 mA g^−1^ based on the weight of LMO. Electrochemical measurements were performed at 25 °C using a HJ1001SD8 battery charge/discharge system (Hokuto Denko Corporation). Charge/discharge capacities were calculated based on the weight of LMO by subtracting the graphene capacity from the observed LMO–G capacity. The graphene capacity in LMO–G was estimated by performing independent electrochemical tests on graphene electrodes under similar charge/discharge conditions.

### Oxidative homocoupling of thiols using LMOs as catalysts

Substrate (0.25 or 1.25 mmol), acetonitrile (1 or 5 mL), LMO (20 mg), and naphthalene (GC internal standard) were placed in a Pyrex glass reactor with a Teflon-coated magnetic stir bar. The reaction mixture was stirred at 0 or 30 °C and ca. 600 rpm under O_2_ or air (1 atm) and LMO was subsequently filtered off using a syringe-filter. Products were quantified by GC and identified by GC-MS.

### Oxidation of sulfides and alkylarenes using LMO(2.3 nm) as a catalyst

LMO(2.3 nm) (50 mg), substrate (0.5 mmol), *o*-dichlorobenzene (1 mL), and naphthalene (GC internal standard) were charged into a PTFE-lined stainless-steel autoclave. The reaction was carried out at 150 °C under O_2_ atmosphere (5 atm) and LMO(2.3 nm) was filtered off upon completion. Products were quantified by GC and identified by GC-MS.

### Oxidative amidation of alcohols using LMO(2.3 nm) as a catalyst

LMO(2.3 nm) (50 mg), substrate (0.25 mmol), 28% aqueous ammonia (50 μL, ca. 2.6 equiv.), 1,4-dioxane (1 mL), and naphthalene (GC internal standard) were charged into a PTFE-lined stainless-steel autoclave. The reaction was carried out at 150 °C under O_2_ atmosphere (3 atm) and LMO(2.3 nm) was filtered off upon completion. Products were quantified by GC and identified by GC-MS.

## Additional Information

**How to cite this article**: Miyamoto, Y. *et al.* Synthesis of ultrasmall Li—Mn spinel oxides exhibiting unusual ion exchange, electrochemical, and catalytic properties. *Sci. Rep.* **5**, 15011; doi: 10.1038/srep15011 (2015).

## Supplementary Material

Supplementary Information
