# Supplementary material for: Synthesis of ultrasmall Li–Mn spinel oxides exhibiting unusual ion exchange, electrochemical, and catalytic properties
Source: Sci Rep. 2015 Oct 12;5:15011. doi: 10.1038/srep15011 (PMC4601032; doi:10.1038/srep15011)
Supplement: Supplementary Information [file srep15011-s1.doc]

*Supplementary Information*

**Synthesis of ultrasmall Li–Mn spinel oxides exhibiting unusual ion exchange, electrochemical, and catalytic properties**

Yumi Miyamoto^1^, Yoshiyuki Kuroda^2^, Tsubasa Uematsu^1^, Hiroyuki Oshikawa^3^, Naoya Shibata^3^, Yuichi Ikuhara^3^, Kosuke Suzuki^1^, Mitsuhiro Hibino^1^, Kazuya Yamaguchi^1^ & Noritaka Mizuno^1^

^1^ Department of Applied Chemistry, School of Engineering, The University of Tokyo, 7-3-1 Hongo, Bunkyo-ku, Tokyo 113-8656, Japan.

^2^ Waseda Institute for Advanced Study, Waseda University, 1-6-1 Nishiwaseda, Shinjuku-ku, Tokyo 169-8050, Japan.

^3^ Institute of Engineering Innovation, School of Engineering, The University of Tokyo, 2-11-16 Yayoi, Bunkyo-ku, Tokyo 113-8656, Japan.

**Supplementary Table 1 |** Li–Mn spinel oxides synthesized by various methods.

| Compound | Synthetic method | Raw materials | Synthetic conditions | Particle size (crystallite size) (nm) | Particle size (direct observation) (nm) | BET surface area (m^2^ g^−1^) | Reference |
| --- | --- | --- | --- | --- | --- | --- | --- |
| LiMn_2_O_4_–RGO | hydrothermal | KMnO_4_, LiOH | 200 °C for 3 h | 3.5 | 10–30 (SEM) | - | 10 |
| LiMn_2_O_4_ | sol-gel (ballmill) | Mn(OAc)_2_, Li(OAc) | 100 °C, 600 °C for 6 h | - | 5 (TEM) | - | S1 |
| LiMn_2_O_4_ | spray pyrolysis | Li(acac), Mn(acac)_3_ | - | 6.8 | - | 203.4 | S2 |
| LiMn_2_O_4_ | template synthesis | Mn_3_O_4_, LiOH | 350 °C for 1 h | - | 7 (wall diameter) (TEM) | 90 | S3 |
| LiMn_2_O_4_ | spray pyrolysis | Li-*t*-butoxide, Mn-2-ethylhexanoate | - | 7 | - | 200 | S4 |
| LiMn_2_O_4_-Graphene (or CNT) | solid state | Mn_3_O_4_, LiOH | 380 °C for 1 h | - | ~7 nm | - | S5 |
| LiMn_2_O_4_ | hydrothermal | LiMnO_2_, LiOH | 180 °C for 12 h | 9 | 15 (average particle size from TEM image) | 38.7 | 5 |
| LiMn_2_O_4_ | hydrothermal | KMnO_4_, LiOH | 180 °C 5 h | 14 | 10–30 (SEM) | - | 38 |
| LiMn_2_O_4_ | spray pyrolysis | Mn(acac)_3_, Li(acac) | 2400–1000 °C 5 min | 10 | - | 134 | S6 |
| LiMn_2_O_4_ | solid state | *α*-MnO_2_, LiOH•H_2_O | 480 °C | - | 10 (diameter) (TEM) | 95.6 | S7 |
| LiMn_2_O_4_ | hydrothermal | Mn(NO_3_)_2_, LiOH | 110 °C for 8 h | - | 10–20 (diameter) (TEM) | 57.85 | S8 |
|  |  |  |  |  |  |  |  |
| LiMn_2_O_4_–CNT | hydrothermal | KMnO_4_, LiOH•H_2_O | 180 °C for 10 h | - | 10–20, 200–500 (SEM) | - | 37 |
| LiMn_2_O_4_ –CNT | hydrothermal | KMnO_4_, LiOH | 180 °C for 5 h | - | 10–20 (diameter) (TEM) | - | 9 |
| LiMn_2_O_4_–RGO | hydrothermal | MnO_2_–RGO, LiOH | 200 °C for 30 min | - | 10–40 (TEM) | - | S9 |
| LiMn_2_O_4_ | sol-gel (ballmill) | Mn(OAc)_2_, Li(OAc) | 205 °C for 1 h, 800 °C for 15 h | 6 | 10 (TEM) | - | S10 |
| LiMn_2_O_4_ | hydrothermal | MnO_2_, LiOH•H_2_O | 180 °C for 96 h | - | 15 (TEM) | - | 35 |
| carbon-coated LiMn_2_O_4_ | hydrothermal | LiOH, Mn(OAc)_2_ | 110 °C for 12 h | - | > 20 (TEM) | 65 | 8 |
| LiMn_2_O_4_ | hydrothermal | Mn(NO_3_)_2_, LiOH | 110 °C for 8 h | - | 20 (diameter)　(TEM) | 57.85 | S11 |
| LiMn_2_O_4_ | solid state | Mn_2_O_3_, LiOH | 700 °C for 10 h | - | 20 (TEM) | 8.6 | 6 |
| LiMn_2_O_4_ | template synthesis | Mn(NO_3_)_2_, Li(NO_3_) | 80 °C for 8 h, 500 °C for 8 h (3 times) | - | 20–30 (wall thickness) (TEM) | 65 | S12 |
| LiMn_2_O_4_ | spray pyrolysis | Mn(OAc)_2_•4H_2_O, Li_2_CO_3_ | - | 20 | 27 (TEM) | - | S13 |
| LiMn_2_O_4_ | hydrothermal | KMnO_4_, LiOH | 180 °C 5 h, 500 °C for 4 h | - | 30–50, 100–300 (SEM) | 68.1 | 36 |
| LiMn_2_O_4_ | combustion synthesis | Mn(NO_3_)_2_, LiNO_3_ | 500 °C for 10 h | - | 40 (TEM) | 3.0252 | S14 |
| LiMn_2_O_4_ | sol-gel | Mn(OAc)_2_•4H_2_O, Li(OAc)•2H_2_O | 450 °C for 5 h, 550 °C for 10 h | 44 | 40–100 (SEM) | 13.81 | S15 |
| LiMn_2_O_4_ | sol-gel | Mn(NO_3_)_2_, Li_2_CO_3_ | 80 °C for 0.5 h, 400 °C | 47 | 50–80 (TEM) | 26 | S16 |
| LiMn_2_O_4_ | combustion synthesis | Mn(NO_3_)•4H_2_O, LiNO_3_ | 500 °C for 15 min | 23 | 50 (SEM) | 3.04 | S17 |
| LiMn_2_O_4_ | molten salt synthesis | Na_0.44_MnO_2_, LiNO_3_, LiCl | 450 °C for 1 h, 800 °C for 1 h | - | 50–100 (diameter) | - | 4 |
| LiMn_2_O_4_ | hydrothermal | MnO_2_ (EMD), Mn(NO_3_)_2_, LiOH•H_2_O | 280 °C for 36 h | - | 50–300 (SEM) | - | S18 |
| LiMn_2_O_4_–CNT | hydrothermal | MnO_2_–CNT, LiOH | 180 °C for 25 h | - | 50–150 (SEM) | - | S19 |
| LiMn_2_O_4_ | sol-gel | Mn(OAc)_2_•4H_2_O, Li(OAc)•2H_2_O | 90 °C for 24 h, 750 °C for 12 h | - | 50–100 (SEM) | 14 | 3 |
| LiMn_2_O_4_ | template synthesis | MnCO_3_, LiI | 70 °C for 12 h, 350 °C for 2 h | - | 60–100 (wall thickness) (TEM) | 78.4 | S20 |
| LiMn_2_O_4_ | sol-gel | Mn(OAc)_2_•4H_2_O, Li(OAc)•2H_2_O | 60 °C, 360 °C for 10 h, 650 °C for 10 h | - | 60 (TEM) | 12.6 | S21 |
| LiMn_2_O_4_ | combustion synthesis | Mn(NO_3_)_2_•6H_2_O, LiNO_3_ | 120 °C, 700 °C | 14–20 | 70 (TEM) | 23 | S22 |
| LiMn_2_O_4_–CNT | hydrothermal | MnO_2_, LiOH | 180 °C for 48 h | - | 100 (SEM) | 16.3 | S23 |
| LiMn_2_O_4_ | sol-gel | Mn(NO_3_)_2_, LiNO_3_ | 110 °C for 12 h, 750 °C for 5 h | - | 100 (TEM) | - | 7 |
| LiMn_2_O_4_ | sol-gel | Mn(OAc)_2_, Li(OAc) | 80 °C for 4 h, 300 °C for 6 h, 800 °C for 6 h | - | <100 (SEM) | - | S24 |
| MgO coated LiMn_2_O_4_ | solid state | MnO_2_, Li(OAc)•2H_2_O | 700 °C for 10 h | - | ca. 100 (SEM) | 18 | S25 |
| LiMn_2_O_4_ | sol-gel | Mn(OAc)_2_, Li(OAc) | 450–700 °C for a few hours | 23 | ca. 100–300 (SEM) | - | S26 |
| LiMn_2_O_4_ | sol-gel | Mn(OAc)_2_, Li(OAc) | 60 °C for 12 h, 600 °C for 10 h | - | 119 (SEM) | ca. 14.5 | S27 |
| LiMn_2_O_4_–CNT | hydrothermal | MnO_2_–CNT, LiOH•H_2_O | 180 °C for 48 h, 700 °C for 8 h | - | 150–400 (TEM) | - | S28 |
| LiMn_2_O_4_ | solid state | Mn(OAc)_2_, Li(OAc) | 750 °C for 6 h | - | 150–500 (TEM) | 1.15 | S29 |
| LiMn_2_O_4_ | solid state | *γ*-MnOOH, LiOH•H_2_O | 750 °C for 3 h | - | ca. 200–300 (SEM) | - | S30 |

RGO: reduced graphene oxide, CNT: carbon nanotube, OAc: acetate, acac: acetylacetonate.

**Supplementary Table 2** | Composition of LMOs with different particle sizes.

| Sample | BET surface area (m^2^ g^−1^) | Particle size (nm)^a^ | Li (wt%) | Mn (wt%) | Li/Mn molar ratio |
| --- | --- | --- | --- | --- | --- |
| LMO(2.3 nm) | 386 | 3.5 | 3.40 | 50.9 | 0.52 |
| LMO(6.7 nm) | 232 | 5.84 | 3.45 | 52.3 | 0.52 |
| LMO(13 nm) | 105 | 12.9 | 3.41 | 53.9 | 0.50 |
| LMO(40 nm) | 16 | 84.7 | 3.23 | 58.0 | 0.44 |
| LMO(bulk) | 3.3 | 410 | 3.83 | 60.8 | 0.50 |

^a^ calculated from the BET surface area

**Supplementary Table 3 |** Li^+^ ion extraction rates, Mn dissolution rates and average oxidation states of Mn in LMO before and after acid treatment.

| Crystallite size (111) (nm) | Li^+^ ion extraction (%) ^a,b^ | Mn dissolution (%)^b^ | Ion exchange reaction (%)^c^ | Average oxidation state of Mn (before acid treatment) | Average oxidation state of Mn (after acid treatment) |
| --- | --- | --- | --- | --- | --- |
| LMO(2.3 nm) | 91.6 | 2.80 | 87.7 | 3.62 ± 0.065 | 3.71 ± 0.042 |
| LMO(6.7 nm) | 94.4 | 6.32 | 73.1 | 3.55 ± 0.071 | 3.69 ± 0.109 |
| LMO(13 nm) | 96.5 | 17.2 | 28.3 | 3.57 ± 0.017 | 3.95 ± 0.044 |
| LMO(40 nm) | 95.9 | 23.9 | 0 | 3.44 ± 0.019 | 3.87 ± 0.043 |
| LMO(bulk) | 89.5 | 19.9 | 0 | 3.48 ± 0.018 | 3.88 ± 0.022 |

^a^ (Li^+^ ion extraction (%)) = (Li/Mn_before acid treatment_ − Li/Mn_after acid treatment_)/(Li/Mn_before acid treatment_) × 100

^b^after stirring LMOs in aqueous nitric solutions at pH 2 for 30 min

^c^ (redox reaction (%)) = (Mn dissolution (%))/[0.25 × (Li^+^ ion extraction (%))] × 100

(ion exchange reaction (%)) = 100 − (redox reaction (%))

**Supplementary Table 4 |** Li^+^ ion extraction from LMO(2.3 nm) and LMO (bulk) under various pH conditions.

| Sample | Reaction conditions | Li^+^ ion extraction (%)^a^ | Mn dissolution (%) | Average oxidation state of Mn |
| --- | --- | --- | --- | --- |
| LMO(2.3 nm) | pH 1 | 94.7 | 4.47 | 3.55 ± 0.028 |
|  | pH 2 | 91.6 | 2.80 | 3.71 ± 0.042 |
|  | pH 3 | 85.7 | 0.740 | 3.60 ± 0.020 |
|  | pH 4 | 66.7 | 0.0100 | 3.62 ± 0.024 |
|  | pH 4^b^ | 84.0 | 0.0400 | - |
|  | pH 5 | 54.6 | 0.0100 | 3.56 ± 0.029 |
|  | pH 5^b^ | 73.2 | 0.0300 | - |
|  | pH 6 | 41.4 | 0.0100 | 3.64 ± 0.022 |
|  | pH 6^b^ | 61.0 | 0.0700 | - |
|  | H_2_O (pH ca. 7) | 18.9 | 0.0400 | 3.59 ± 0.070 |
|  | H_2_O (pH ca. 7)^b^ | 29.6 | 0.190 | - |
|  | before acid treatment | - | - | 3.62 ± 0.065 |
| LMO(bulk) | pH 1 | 90.0 | 19.8 | 3.78 ± 0.032 |
|  | pH 2 | 89.5 | 19.9 | 3.88 ± 0.022 |
|  | pH 3 | 55.5 | 13.2 | 3.59 ± 0.047 |
|  | pH 4 | 42.1 | 10.2 | 3.53 ± 0.061 |
|  | pH 4^b^ | 51.2 | 11.7 | - |
|  | pH 5 | 5.77 | 1.76 | 3.44 ± 0.040 |
|  | pH 5^b^ | 8.67 | 1.87 | - |
|  | pH 6 | 0.00 | 0.380 | 3.44 ± 0.014 |
|  | pH 6^b^ | 5.02 | 0.330 | - |
|  | H_2_O (pH ca. 7) | 0.00 | 0.0100 | 3.41 ± 0.039 |
|  | H_2_O (pH ca. 7)^b^ | 1.67 | 0.0100 | - |
|  | before acid treatment | - | - | 3.48 ± 0.018 |

^a^ (Li^+^ ion extraction (%)) = (Li/Mn_before acid treatment_ − Li/Mn_after acid treatment_)/(Li/Mn_before acid treatment_) × 100

^b^after two Li^+^ ion extraction cycles

**Supplementary Table 5 |** Li^+^ ion extraction from LMO using a large amount of aqueous nitric acid solution^a^.

|  | Li^+^ ion extraction (%)^b^ | | Mn dissolution (%) | | Ion-exchange-type reaction (%)^c^ | |
| --- | --- | --- | --- | --- | --- | --- |
|  | LMO  (2.3 nm) | LMO  (bulk) | LMO  (2.3 nm) | LMO  (bulk) | LMO  (2.3 nm) | LMO  (bulk) |
| pH4 | 94.1 | 88.6 | 1.95 | 22.2 | 91.7 | 0 |
| pH5 | 89.5 | 44.9 | 0.290 | 10.3 | 98.7 | 0 |

^a^1000 mL aqueous nitric acid solution for 10 mg Li–Mn spinel oxide

^b^(Li^+^ ion extraction (%)) = (Li/Mn_before acid treatment_ − Li/Mn_after acid treatment_)/(Li/Mn_before acid treatment_) × 100

^c^(redox reaction (%)) = (Mn dissolution (%))/[0.25 × (Li^+^ ion extraction (%)] × 100

(ion exchange reaction (%)) = 100 − (redox reaction (%))

**Supplementary Table 6 |** Composition of -MnO_2_ nanoparticles obtained by Li^+^ ion extraction from LMO(2.3 nm) before and after dispersion in 0.1 M LiCl−LiOH aqueous solutions.

| Reaction time | Li/Mn molar ratio |
| --- | --- |
| Before dispersion | 0.047 |
| 30 min | 0.32 |
| 6 h | 0.35 |

**Supplementary Table 7 |** Synthetic conditions using 2-propanol/H_2_O mixtures.

| H_2_O/Li molar ratio | 2-propanol (mL) | H_2_O (mL) | LiCl (mmol) | TBAMnO_4_ (mmol) |
| --- | --- | --- | --- | --- |
| 10 | 3.2 | 1.8 | 10 | 0.15 |
| 20 | 1.4 | 3.6 | 10 | 0.15 |
| 200 | 0.5 | 4.5 | 1.25 | 0.15 |
| 500 | 5.0 | 45 | 5 | 1.5 |

**Supplementary Table 8 |** Cathode compositions.

| Active material | LMO (wt%) | Graphene (wt%) | Acetylene black (wt%) | PTFE (wt%) |
| --- | --- | --- | --- | --- |
| LMO–G | 15 | 30 | 45 | 10 |
| LMO(2.3 nm) | 46 | - | 47 | 7 |
| LMO(6.7 nm) | 46 | - | 47 | 7 |
| LMO(13 nm) | 46 | - | 47 | 7 |
| LMO(40 nm) | 47 | - | 47 | 6 |
| LMO(bulk) | 49 | - | 45 | 6 |


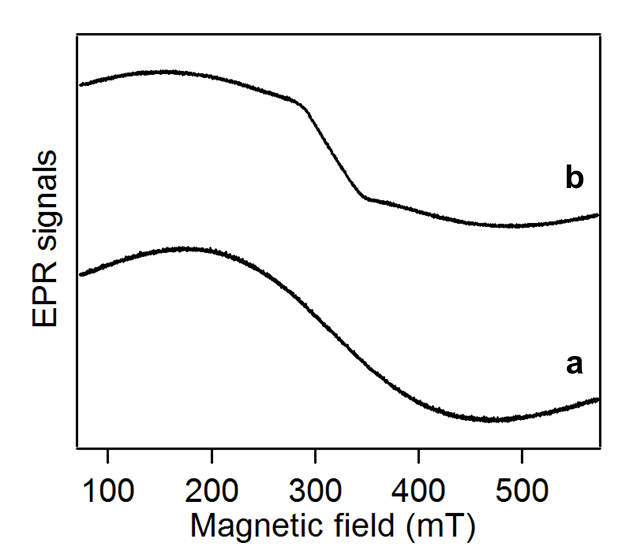


**Supplementary Figure 1 |** EPR spectra of LMO(2.3 nm) (**a**) before and (**b**) after stirring in aqueous nitric acid solutions at pH 2 for 30 min.

**
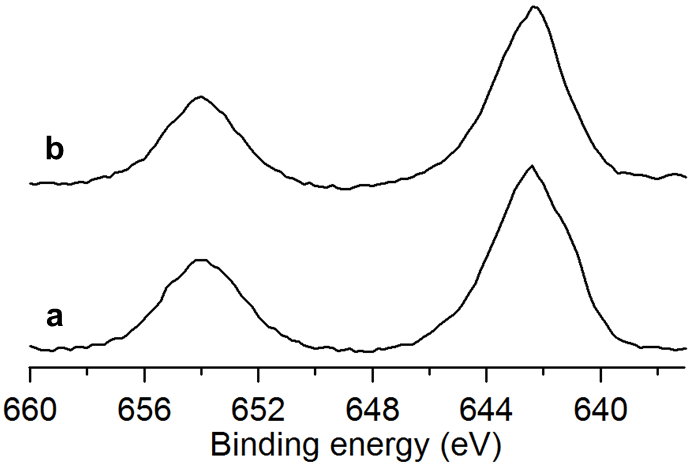
**

**Supplementary Figure 2 |** XPS spectra of (**a**) LMO(bulk) and (**b**) LMO(2.3 nm) in the Mn 2p regions.


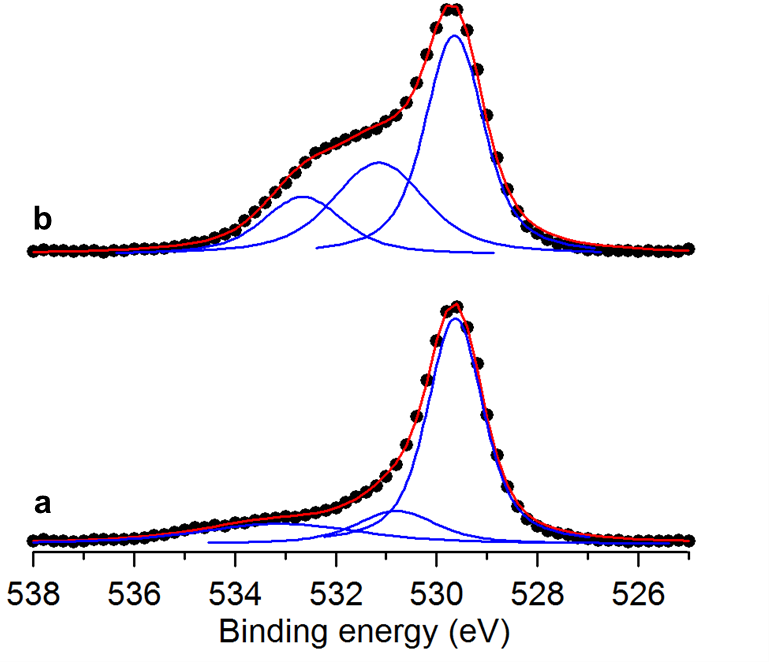


**Supplementary Figure 3 |** XPS spectra of (**a**) LMO(bulk) and (**b**) LMO(2.3 nm) in the O 1s regions. The filled circles show the experimental XPS spectra of LMOs. The blue and red lines show the deconvolution of the spectra and the sum of the deconvolution, respectively. The low (529.6 eV), medium (530.8–531.1 eV), and high binding energy peaks (532.7–533.1 eV) show the lattice oxygen, the surface adsorbed oxygen or OH groups on the surface and oxygen vacancies, and adsorbed molecular H_2_O, respectively^48^.


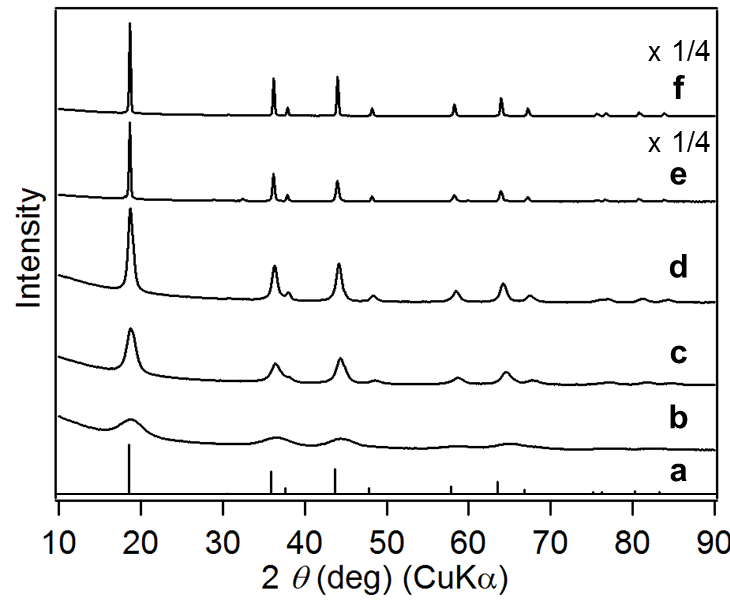


**Supplementary Figure 4 |** (**a**) Literature data of LiMn_2_O_4_ (JCPDS 35-0782). XRD patterns of LMOs with different crystallite sizes (**b**) LMO(2.3 nm), (**c**) LMO(6.7 nm), (**d**) LMO(13 nm), (**e**) LMO(40 nm), (**f**) LMO(bulk).

**Supplementary Figure 5 |** XRD patterns of (**a**) LMO(2.3 nm), (**b**) LMO(6.7 nm), (**c**) LMO(13 nm), (**d**) LMO(40 nm), (**e**) LMO(bulk) before and after stirring in aqueous nitric acid solutions at pH 2 for 30 min.

**Supplementary Figure 6 |** XRD patterns of (**A**) LMO(2.3 nm) and (**B**) LMO(bulk) after stirring in aqueous nitric acid solutions for 30 min under various pH conditions ((**a**) pH 1, (**b**) pH 2, (**c**) pH 3, (**d**) pH 4, (**e**) pH 5, (**f**) pH 6, (**g**) pH ~7 (H_2_O), and (**h**) before acid treatment). For (**g**), LMOs were only stirred for 30 min in deionized water without pH adjustment.

**Supplementary Figure 7 |** XRD patterns of (**A**) LMO(2.3 nm) and (**B**) LMO(bulk) after two acid treatment cycles under various pH conditions for 30 min ((**a**) pH 4, (**b**) pH 5, (**c**) pH 6, and (**d**) pH ~7 (H_2_O)). For (**d**), Li–Mn spinel oxides were only stirred for 30 min in deionized water without pH adjustment.

**Supplementary Figure 8 |** XRD patterns of (**A**) LMO(2.3 nm) and (**B**) LMO(bulk) after stirring in large amount of aqueous nitric acid solution (1000 mL aqueous nitric acid solution for 10 mg Li–Mn spinel oxide) at (**a**) pH 4 and (**b**) pH 5.

**Supplementary Figure 9 |** XRD patterns of λ-MnO_2_ nanoparticles obtained by Li^+^ ion extraction from LMO(2.3 nm) (**a**) before and after dispersion in 0.1 M LiCl–LiOH aqueous solutions for (**b**) 30 min, and (**c**) 6 h.

**Supplementary Figure 10 |** (**a**) Models of particles with various sizes, (**b**) nanoparticle site energy, and (**c**) simulated chemical potential change curves of particles involving different volume fractions of the particle surface region.

**Supplementary Figure 11 |** SEM image of LMO(2.3 nm).


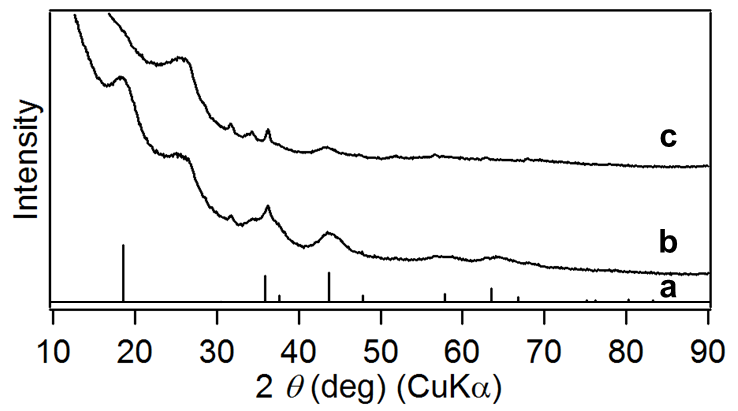


**Supplementary Figure 12 |** (**a**) Literature data of LiMn_2_O_4_ (JCPDS 35-0782). XRD patterns of (**b**) LMO–G and (**c**) graphene.

**Supplementary Figure 13 |** XRD patterns of LMO(2.3 nm) used as catalysts for the homocoupling of cyclohexanethiol (**a**) before use and after (**b**) first and (**c**) second reuse. Reaction conditions: LMO(2.3 nm) (20 mg), substrate (0.25 mmol), acetonitrile (1 mL), 30 °C, O_2_ (1 atm), 1 min.

**Supplementary References**

S1. Kamarulzaman, N. *et al*. Investigation of cell parameters, microstructures and electrochemical behaviour of LiMn_2_O_4_ normal and nano powders. *J. Power Sources* **188**, 274–280 (2009).

S2. Patey, T. J., Büchel, R., Nakayama, M. & Novák, P. Electrochemistry of LiMn_2_O_4_ nanoparticles made by flame spray pyrolysis. *Phys. Chem. Chem. Phys.* **11**, 3756–3761 (2009).

S3. Jiao, F., Bao, J., Hill, A. H. & Bruce, P. G. Synthesis of ordered mesoporous Li–Mn–O spinel as a positive electrode for rechargeable lithium batteries. *Angew. Chem. Int. Ed.* **47**, 9711–9716 (2008).

S4. Ernst, F. O. *et al*. Electrochemically active flame-made nanosized spinels: LiMn_2_O_4_, Li_4_Ti_5_O_12_ and LiFe_5_O_8_. *Mater. Chem. Phys*. **101**, 372–378 (2007).

S5. Zhao, X., Hayner, C. M. & Kung, H. H. Self-assembled lithium manganese oxide nanoparticles on carbon nanotube or graphene as high-performance cathode material for lithium-ion batteries. *J. Mater. Chem.* **21**, 17297–17303 (2011).

S6. Chew, S. Y. *et al*. Thin nanostructured LiMn_2_O_4_ films by flame spray deposition and in situ annealing method. *J. Power Sources* **189**, 449–453 (2009).

S7. Lee, H.–W. *et al*. Ultrathin spinel LiMn_2_O_4_ nanowires as high power cathode materials for Li-ion batteries. *Nano Lett.* **10**, 3852–3856 (2010).

S8. Zhang, Q.–H., Li, S.–P., Sun, S.–Y., Yin, X.–S. & Yu, J.–G. LiMn_2_O_4_ spinel direct synthesis and lithium ion selective adsorption. *Chem. Eng. Sci.* **65**, 169–173 (2010).

S9. Bak, S.–M. *et al.* Spinel LiMn_2_O_4_/reduced graphene oxide hybrid for high rate lithium ion batteries. *J. Mater. Chem*. **21**, 17309–17315 (2011).

S10. Singhal, R., Resto, O. & Katiyar, R. S. Effect of nanocrystallinity on the electrochemical performance of LiMn_2_O_4_ cathode. *J. Renewable Sustainable Energy* **1**, 023103 (2009).

S11. Zhang, Q.–H., Sun, S.–Y., Li, S.–P., Yin, X.–S. & Yu, J.–G. Direct hydrothermal synthesis of ternary Li–Mn–O oxide ion-sieves. *Ann. N. Y. Acad. Sci.* **1161**, 500 (2009).

S12. Li, X., Cheng, F., Guo, B. & Chen, J. Template-synthesized LiCoO_2_, LiMn_2_O_4_, and LiNi_0.8_Co_0.2_O_2_ nanotubes as the cathode materials for lithium ion batteries. *J. Phys. Chem. B* **109**, 14017–14024 (2005).

S13. Yi, J. H. *et al.* Nanosized LiMn_2_O_4_ powders prepared by flame spray pyrolysis from aqueous solution. *J. Power Sources* **196**, 2858–2862 (2011).

S14. Lu, C.–Z. & Fey, G. T.–K. Nanocrystalline and long cycling LiMn_2_O_4_ cathode material derived by a solution combustion method for lithium ion batteries. *J. Phys. Chem.* **67**, 756–761 (2006).

S15. Chen, Y., Xie, K., Pan, Y. & Zheng, C. Effect of calcination temperature on the electrochemical performance of nanocrystalline LiMn_2_O_4_ prepared by a modified resorcinol–formaldehyde route. *Solid State Ionics* **181**, 1445–1450 (2010).

S16. Choy, J.–H., Kim, D.–H., Kwon, C.–W., Hwang, S.–J. & Kim, Y.–I. Physical and electrochemical characterization of nanocrystalline LiMn_2_O_4_ prepared by a modified citrate route. *J. Power Sources* **77**, 1–11 (1999).

S17. Fey, G. T.–K., Cho, Y.–D. & Kumar, T. P. Nanocrystalline LiMn_2_O_4_ derived by HMTA-assisted solution combustion synthesis as a lithium-intercalating cathode material. *Mater. Chem. Phys.* **99**, 451–458 (2006).

S18. Wu, H. M. *et al*. One-step synthesis LiMn_2_O_4_ cathode by a hydrothermal method. *J. Power Sources* **161**, 1260–1263 (2006).

S19. Ding, Y., Li, J., Zhao, Y. & Guan, L. Direct growth of LiMn_2_O_4_ on carbon nanotubes as cathode materials for lithium ion batteries. *Mater. Lett*. **68**, 197–200 (2012).

S20. Wu, Y., Wen, Z., Feng, H. & Li, J. Hollow porous LiMn_2_O_4_ microcubes as rechargeable lithium battery cathode with high electrochemical performance. *Small* **8**, 858–862 (2012).

S21. Chen, Y., Xie, K., Pan, Y. & Zheng, C. Nano-sized LiMn_2_O_4_ spinel cathode materials exhibiting high rate discharge capacity for lithium-ion batteries. *J. Power Sources* **196**, 6493–6497 (2011).

S22. Raja, M. W., Mahanty, S., Ghosh, P., Basu, R. N. & Maiti, H. S. Alanine-assisted low-temperature combustion synthesis of nanocrystalline LiMn_2_O_4_ for lithium-ion batteries. *Mater. Res. Bull.* **42**, 1499–1506 (2007).

S23. Zou, B.–K. *et al*. High rate LiMn_2_O_4_/carbon nanotube composite prepared by a two-step hydrothermal process. *J. Power Sources* **268**, 491–497 (2014).

S24. Hwang, B. J., Santhanam, R. & Liu, D. G. Characterization of nanoparticles of LiMn_2_O_4_ synthesized by citric acid sol-gel method. *J. Power. Sources* **97–98**, 443–446 (2001).

S25. Lim, S. & Cho, J. PVP-functionalized nanometre scale metal oxide coatings for cathode materials: successful application to LiMn_2_O_4_ spinel nanoparticles. *Chem. Commun.* 4472–4474 (2008).

S26. Michalska, M. Chemical syntheses of nanocrystalline lithium manganese oxide spinel. *Phys. Status. Solidi C* **8**, 2538–2541 (2011).

S27. Murugan, A. V., Kale, B. B., Kunde, L. B. & Kulkarni, A. V. Comparison of different soft chemical routes synthesis of nanocrystalline LiMn_2_O_4_ and their influence on its physicochemical properties. *J. Solid State Electrochem*. **10**, 104–109 (2006).

S28. Tang, M., Yuan, A., Zhao, H. & Xu, J. High-performance LiMn_2_O_4_ with enwrapped segmented carbon nanotubes as cathode material for energy storage. *J. Power Sources* **235**, 5–13 (2013).

S29. Sakunthala, A., Reddy, M. V., Selvasekarapandian, S., Chowdari, B. V. R. & Selvin, P. C. Synthesis of compounds, Li(MMn_11/6_)O_4_ (M = Mn_1/6_, Co_1/6_, (Co_1/12_Cr_1/12_), (Co_1/12_Al_1/12_), (Cr_1/12_Al_1/12_)) by polymer precursor method and its electrochemical performance for lithium-ion batteries. *Electrochim. Acta* **55**, 4441–4450 (2010).

S30. Zhao, H. *et al.* A novel two-step preparation of spinel LiMn_2_O_4_ nanowires and its electrochemical performance characterization. *J. Mater. Res.* **27**, 1750–1754 (2012).
